# Antiretroviral resistance, genotypic characterization and origin of Human Immunodeficiency Virus among the infected wives of Intravenous drug users in Manipur

**DOI:** 10.1038/s41598-018-33636-z

**Published:** 2018-10-12

**Authors:** Adhikarimayum Lakhikumar Sharma, Thiyam Ramsing Singh, Lisam Shanjukumar Singh

**Affiliations:** Cancer and Molecular Biology Division, Department of Biotechnology, Manipur University, Canchipur, 795003 Imphal, Manipur India

## Abstract

Increasing incidence of drug resistance is ascertained to be the main obstacles in limiting the virus among the human immunodeficiency virus (HIV) infected individuals. This study investigates the drug resistance mutations (DRMs), genetic variants and origin of transmitted drug resistance of HIV-1 among the HIV-1 infected wives of intravenous drug users (IDUs) in Manipur. 44 HIV *pol* gene sequences were generated from 56 blood samples by viral gene amplification and sequencing. Sequences were then analysed for drug resistance, genetic variants and origin. The result revealed that among the treatment naive cases, 35.7% had Transmitted Drug Resistance Mutations (TDRMs) while among treatment experienced cases, 50% had Acquired Drug Resistant Mutations (ADRMs). These TDRMs and ADRMs conferred resistance to nucleoside reverse transcriptase inhibitors (NRTIs), non-nucleoside reverse transcriptase inhibitors (NNRTIs) and/or protease inhibitors (PIs). Majority of the isolated HIV-1 sequences (77.3%) were subtype C while 9.1% was discordant subtype, 6.8% was subtype B, 4.5% was CRF_01AE and 2.3% was URF_BC. TDRM strains were found to be introduced from Myanmar, Vietnam and mainland India. This study also reveals the appearance of CRF_01AE for the first time in Manipur. The finding of this study indicates high prevalence of drug resistant mutations and complex molecular epidemiology in Manipur.

## Introduction

HIV/AIDS in Manipur, northeast India has emerged as a serious public health emergency ever since it was first detected in the year 1989. According to the National AIDS Control Organization (NACO), India in the year 2015, Manipur has the highest (1.15%) adult HIV prevalent state in the country^1^. Infection with HIV/AIDS in Manipur is rapidly spreading. Manipur, which lies between the mainland India and the golden triangle of South-East Asia, the second largest opium producer in the world, has been dominated by HIV infected IDUs group^2–4^. The severity of HIV/AIDS pandemic is associated with IDUs and it remains a burden to the state^5^. The reason for high number of IDUs was explained due to the easy availability of heroin through illegal drug trafficking from golden triangle area^6^. The IDUs share the injecting equipment among them and hence enhance the chance of contamination with HIV and other blood borne viral infections like Hepatitis B and C infection^2^. However, the current scenario shows the clear expansion of the HIV/AIDS cases to the wives and children of IDUs^7^. The transmissions of HIV from IDUs to their wives have shown that 45% wives were HIV positive^8^. Moreover, the seroprevalence of HIV infection among the housewives and pregnant women had increased^9^. To limit the expansion of HIV, various programs had been initiated in India. NACO initiated ART program countrywide on 1st April 2004 and to intensify the program, the second line of ART program was started in January 2008. Even though, ART is now available free of cost, the treatment is initiated depending upon the CD4 count as indication of staging of HIV infection. PLHIV with CD4 count less than 200 blood cells/mm^3^ require treatment irrespective of the clinical stage. Patients on ART need to be constantly monitored for their response to ART drugs treatment with clinical, immunological and virological criteria. The reasons for monitoring the effectiveness of ART are to measure ART adherence, to guide the patient for timely switching to second-line ART, to avoid severe clinical outcomes, and to prevent onward HIV transmission including resistant virus^10^. Previously we have conducted a study to investigate the HIV-1 drug resistance among the ART experienced HIV individual in the region to understand the genetic basis of drug resistance. The data revealed that 53% of HIV-1 infected individuals residing in Manipur harbored drug resistance mutations at different drug resistance positions^11^. Nevertheless, there is no data on the wives of IDUs even though there is clear evidence of HIV/AIDS expansion from IDUs to general population through them. Wives of IDUs are gradually accounting for a larger proportion of HIV infections and ultimately becoming the new face of the HIV epidemic. Therefore, this current study is conducted to investigate the TDR and ADR (to the NRTIs, NNRTIs and PIs drug classes), genetic variant and origin of the TDR among the HIV-1 infected wives of IDUs residing in Manipur state during the year 2016–2017.

## Results

### Clinical characteristics of study participants

Table 1 show the clinical characteristics of participants. 56 HIV-1 infected wives of IDUs were enrolled for the study during August 2016 and September 2017 (Supplementary Table S1). All of them were infected with HIV from their spouses probably through sexual mode. Among the 56 recruited individuals, 18 had never been experienced to ART drugs while remaining 38 individuals had experienced ART for more than 6 months to the following ART drug combinations at the time of sample collection: ZLN; Zidovudine + lamivudine + nevirapine, (AZT + 3TC + NVP) and TLE; Tenofovir + Lamivudine + efavirenz (TDF + 3TC + EFV) as a fixed-dose combination but none started a PI-based regimen. The CD4 T cell counts of the participants were ≤200 cells/μl for 14.3%, between 201 and 400 cells/μl for 48.2%, and ≥401cells/μl for 37.5%. The average CD4 count among the ART naïve and ART experienced individuals were 220 cells/μl and 350 cells/μl respectively. The percentage of each category of particular factors (age, CD4 counts, year of HIV diagnose, ART treatment status, participants’ level of education, etc.) is summarized in Table 1. From the 56 blood samples, a total of 44 samples were amplified and sequenced with a success rate of 78% (44/56). The failure of PCR amplification or sequencing (22%) might be due to the degradation of samples, poor transportation and/or sequencing primer specificity.

**Table 1:** Clinical characteristics of HIV-1 infected wives of IDUs.

Samples	n = 56 (%)
Age (median)	33 years
**CD4 count (cells/μl)**	
≤200	08 (14.3%)
201–400	27 (48.2%)
≥401	21 (37.5%)
**Average CD4 based on ART status**	
ART naive	220 cells/µl
ART experienced	350 cells/µl
**HIV-1 diagnose year**	
2014	10 (17.9%)
2015	24 (42.8%)
2016	22 (39.3%)
**ART Treatment status**	
Naïve	18 (32.1%)
Experienced (AZT + 3TC + NVP/TDF + 3TC + EFV) (For more than 6 months)	38 (67.9%)
**Employment**	
Govt employee	05 (8.9%)
Private employee	10 (17.9%)
Unemployed	41 (73.2%)
**Education**	
Below 10 standard	21 (37.5%)
Graduate	33 (58.9%)
Post graduate	02 (3.6%)

“n” denotes the sample size of the study.

### HIV-1 genetic characterization

**Figure 1:**
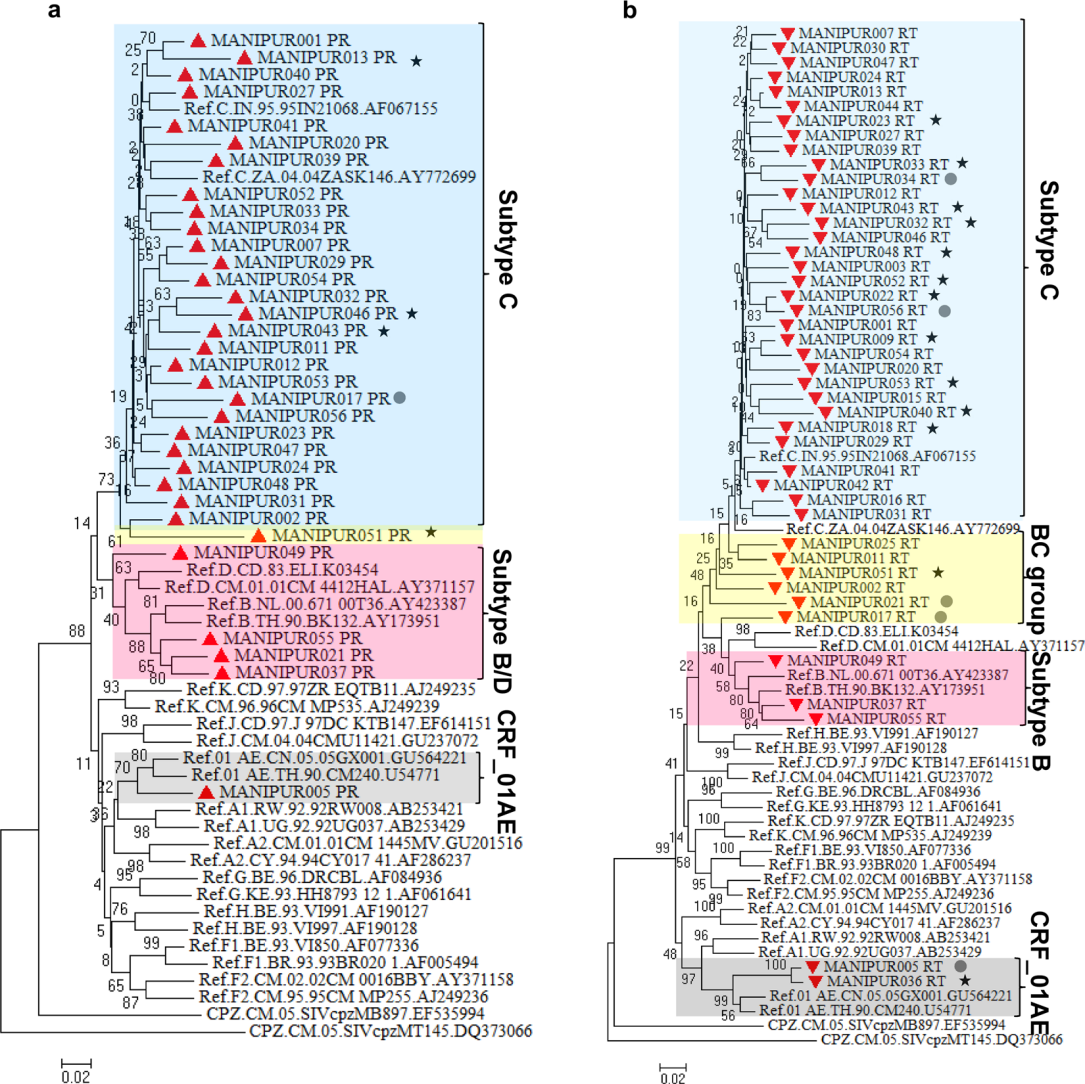
Phylogenetic Neighbor-Joining tree of HIV *pol* gene sequences of HIV infected wives of IDUs in Manipur. Phylogenetic analysis of HIV-1 protease (PR) and reverse transcriptase (RT) gene sequences. Phylogenetic trees were generated from the sequenced HIV-1 PR (**a**) and RT (**b**) genes together with the corresponding viral gene of reference. The tree was inferred using the Neighbor-Joining method. The optimal tree with the sum of branch length = 2.5 is shown. The tree was computed using the Kimura 2-parameter method and are in the units of the number of base substitutions per site. The rate variation among sites was modeled with a gamma distribution (shape parameter = 1). The phylogenetic trees analyses were conducted in MEGA7. Manipur sequences were indicated with solid red triangle, Drug resistance of the ART naïve and ART treatment experienced individuals were represented with solid circle and solid stars respectively.

44 *pol* nucleotide sequences (33 PR and 44 RT sequences) were obtained from 56 blood samples by viral gene amplification, sequencing. The newly generated sequences were submitted to the GenBank under GenBank accession numbers: MG251453-MG251529. Out of the 44 *pol* nucleotides sequences, 14 were obtained from treatment naïve individuals. Using either PR and/or RT regions of HIV *pol* gene, 44 samples were genotyped successfully. Based on the phylogenetic bootstrap consensus tree constructed with 33 PR sequences and reference sequences, four samples Manipur021_PR, Manipur037_PR, Manipur049_PR and Manipur055_PR formed cluster with subtype B group, one sample Manipur005_PR with CRF_01AE and another sample Manipur051_PR was identified as recombinant BC while the rest of samples were cluster with subtype C (Fig. 1a). The analysis of independent phylogenetic trees of 44 RT sequences revealed that two samples (Manipur005_RT and Manipur036_RT) were CRF_01AE, 3 samples (Manipur037_RT, Manipur049_RT and Manipur055_RT) were formed cluster with subtype B, and five samples were intersubtype recombination BC while remaining sequences were identified as subtype C (Fig. 1b). This result is consistent when the samples were genotyped with REGA/COMET. BC intersubtype recombination and CRF_01AE at RT gene were also confirmed by RIP, jPHMM (Supplementary Fig S1) and bootscanning in Simplot software (Supplementary Fig. S2).

**Figure 2:**
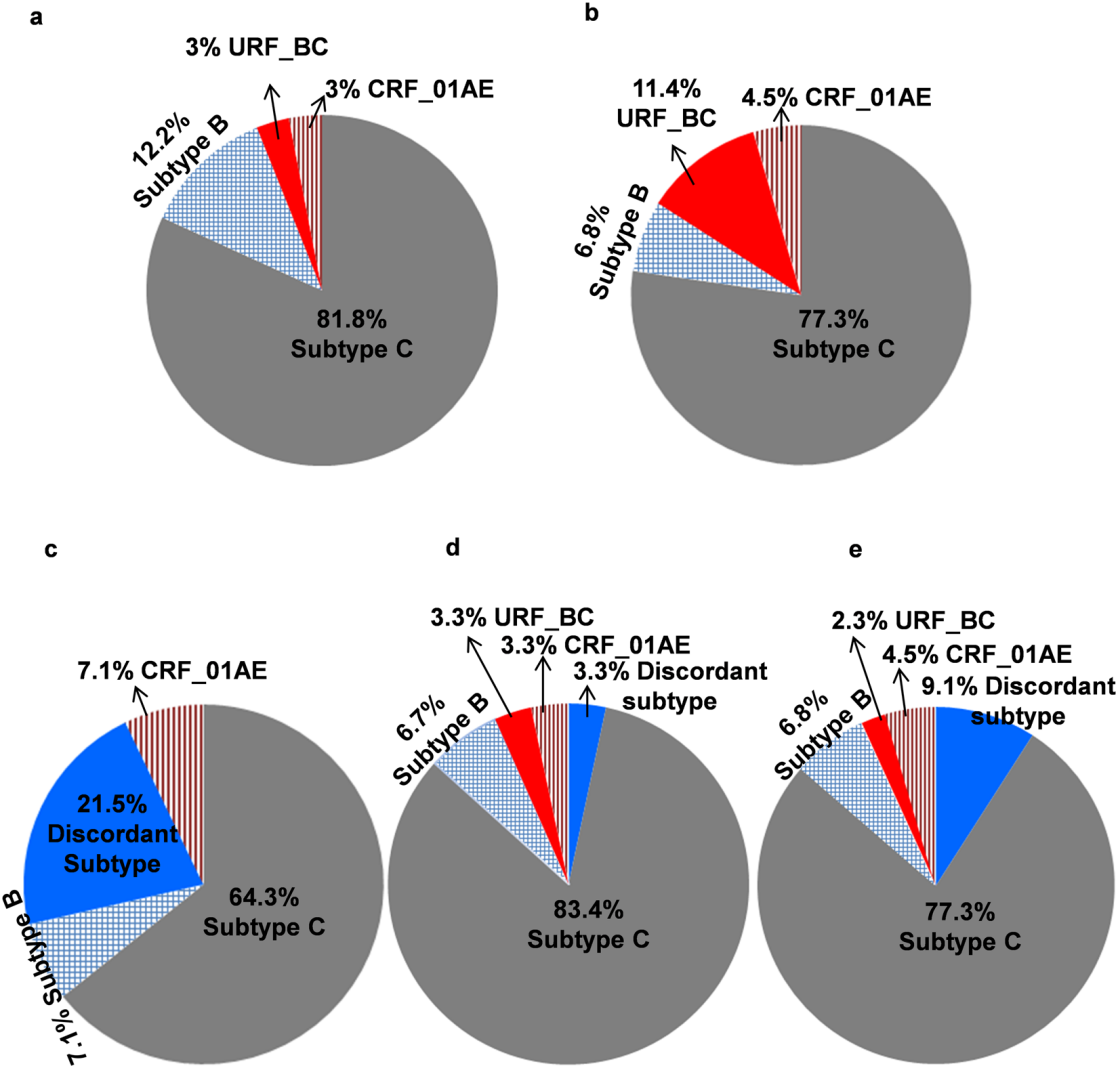
Pie chart represents the distribution of HIV-1 Group M clades and recombinant forms of viral subtypes among the HIV infected wives of IDUs. Viral subtypes according to protease (**a**), reverse transcriptase (**b**), ART naïve individuals (**c**), ART treatment experienced individuals (**d**) and final subtypes of study participants (**e**).

The analysis of PR region revealed that 81.8% was subtype C, 3% was URF_BC, 12.2% was subtype B and 3% was CRF_01AE while analysis of RT region showed that 77.3% was subtype C, 11.4% was URF_BC, 6.8% was subtype B and 4.5% was CRF_01AE (Fig. 2a,b).

Among the 14 treatment naïve individuals, nine individuals (64.3%) harbored subtype C strain, 3 individuals (21.5%) harbored discordant subtype virus, and one each individual’s (7.1% each) harbored subtype B and CRF_01AE viral strain (Fig. 2c). Among the 30 treatment experienced individuals, 83.3% was subtype C, 6.7% was subtype B and 3.3% each was URF_BC, CRF_01AE and discordant subtype (Fig. 2d).

Finally, based on PR and RT regions, 34 out of 44 samples (77.3%) were subtype C group while 4 (9.1%) were discordant subtype, 3 (6.8%) were subtype B, 2 (4.5%) were CRF_01AE and 1 (2.3%) was URF_BC (Fig. 2e) (Table 2).

**Table 2:** Genetic variant in *pol* gene of HIV-1 among the infected wives of IDUs.

SAMPLE	PR GENE	RT GENE	FINAL SUBTYPE
MANIPUR001	C	C	C
MANIPUR002	C	BC	Discordant subtypes
MANIPUR003	NA	C	C
MANIPUR005	CRF_01AE	CRF_01AE	CRF_01AE
MANIPUR007	C	C	C
MANIPUR009	NA	C	C
MANIPUR011	C	BC	Discordant subtypes
MANIPUR012	C	C	C
MANIPUR013	C	C	C
MANIPUR015	NA	C	C
MANIPUR016	NA	C	C
MANIPUR017	C	BC	Discordant subtypes
MANIPUR018	NA	C	C
MANIPUR020	C	C	C
MANIPUR021	B	BC	Discordant subtypes
MANIPUR022	NA	C	C
MANIPUR023	C	C	C
MANIPUR024	C	C	C
MANIPUR025	NA	C	C
MANIPUR027	C	C	C
MANIPUR029	C	C	C
MANIPUR030	NA	C	C
MANIPUR031	C	C	C
MANIPUR032	C	C	C
MANIPUR033	C	C	C
MANIPUR034	C	C	C
MANIPUR036	NA	CRF-01AE	CRF_01AE
MANIPUR037	B	B	B
MANIPUR039	C	C	C
MANIPUR040	C	C	C
MANIPUR041	C	C	C
MANIPUR042	NA	C	C
MANIPUR043	C	C	C
MANIPUR044	NA	C	C
MANIPUR046	C	C	C
MANIPUR047	C	C	C
MANIPUR048	C	C	C
MANIPUR049	B	B	B
MANIPUR051	BC	BC	URF_BC
MANIPUR052	C	C	C
MANIPUR053	C	C	C
MANIPUR054	C	C	C
MANIPUR055	B	B	B
MANIPUR056	C	C	C

The final subtype was assigned based on HIV subtyping tools; REGA (version 3.0)/jpHMM, COMET and phylogenetic tree. Recombination was further confirmed by Recombinant Identification Program (RIP) and bootstrapping in Simplot software 3.5.1. NA, not available; PR, protease; RT, reverse transcriptase.

### Transmitted and acquired drug resistance

**Figure 3:**
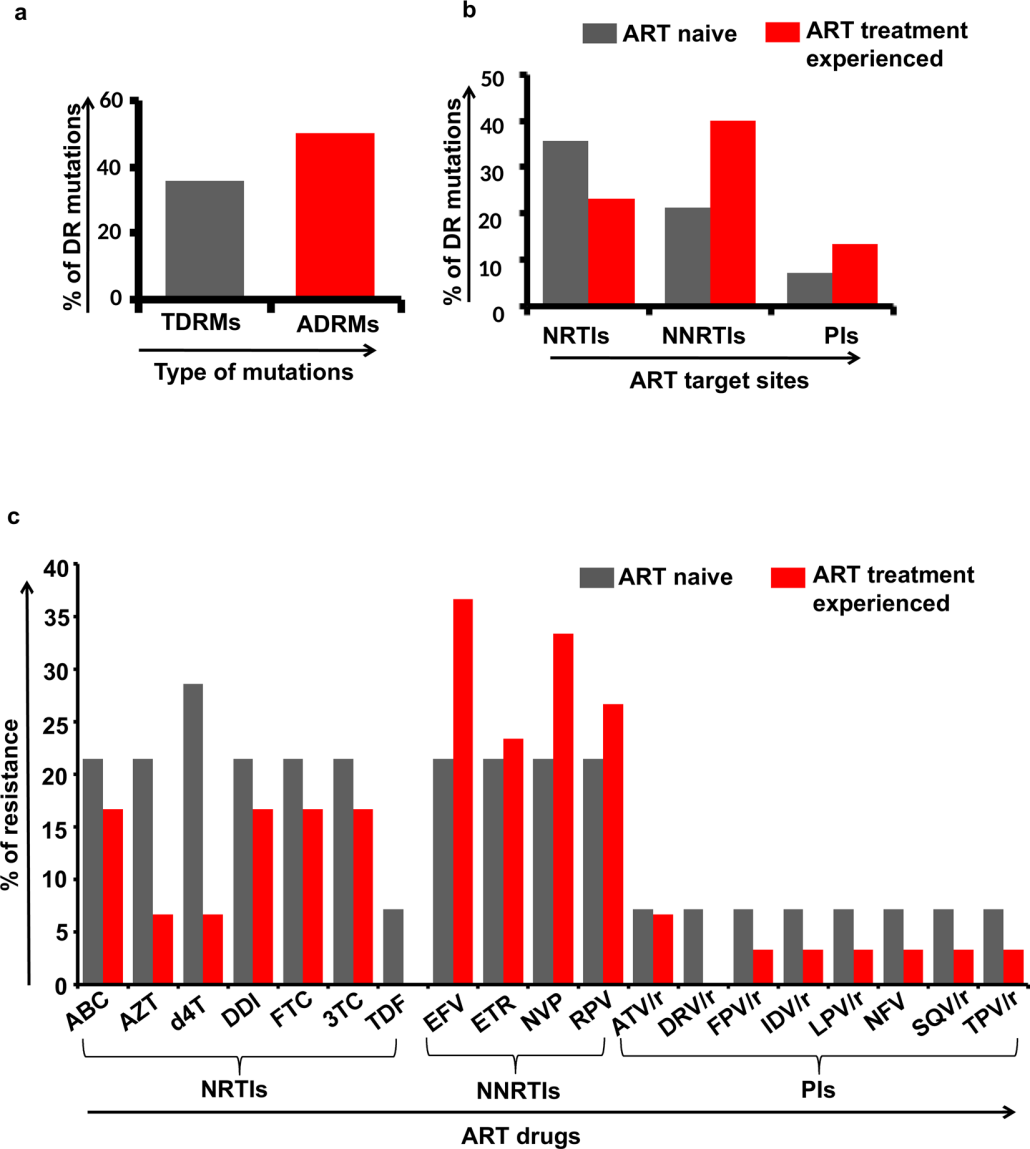
Graphical representation of drug resistance mutations and resistance against different antiretroviral drugs; Transmitted and acquired drug resistant mutation of HIV-1 (**a**), drug resistance mutation among the ART naïve and ART treatment experienced individuals at NRTIs, NNRTIs and PIs target sites (**b**) and percentage of confer resistance against the ART drugs among the ART naïve individuals and the ART treatment experienced individuals (**c**).

The analysis of drug resistance mutations revealed that among the 14 treatment-naïve patients analyzed in the study population, five patients (35.7%, 5/14) harbored TDRMs (Fig. 3a). Further, 5 (37.5%, 5/14), 3 (21.4%, 3/14) and 1 (7.1%, 1/14) patients have mutations at the target sites for NRTIs, NNRTIs and PIs respectively (Fig. 3b). Nine different types of TDRMs in five individuals have been defined based on the mutation sites (5 at NRTIs and 4 at NNRTIs target sites) in the reverse transcriptase gene associated with resistance to reverse transcriptase inhibitors and three different types of TDRMs in protease gene associated with resistance to protease inhibitors. One sample, Manipur017 harbored multiple mutations in NRTIs (K65R, M184V), NNRTIs (Y188L) and PIs (I54V, L76V and I84V) which conferred high-level resistance to abacavir (ABC), didanosine (DDI), emtricitabine (FTC), lamivudine (3TC), and intermediate-level resistance to stavudine (d4T), tenofovir (TDF), high or intermediate level resistance to all NNRTIs and PIs (Table 3). The common DRMs found among the ART naïve patients were targeted at (i) NRTI positions: M184V (21.4%, 3/14), K219Q (14.2%, 2/14) and K65R, Y115F and T215F (7.1% each, 1/14) (ii) NNRTI positions: Y181C (14.2%, 2/14) and K103N, G190A and Y188L (7.1% each, 1/14) and (iii) protease inhibitors positions: I54V, L76V and I84V (7.1% each,1/14). Further, our study also reveals that 21.4% of the individuals was found to be harbored virus which has the capability to confer resistance to NRTIs and NNRTIs drugs; ABC, AZT, DDI, FTC, 3TC, efavirenz (EFV), etravirine (ETR), nevirapine (NVP) and rilpivirine (RPV) and 28.6% individual to d4T while 7.1% of the individuals was found to be harbored virus which has the capability to confer resistance to PI (protease inhibitors drugs) drugs; atazanavir (ATV/r), darunavir (DRV/r), fosamprenavir (FPV/r), indinavir (IDV/r), lopinavir (LPV/r), nelfinavir (NFV), saquinavir (SQV/r) and tipranavir (TPV/r) (Fig. 3c).

**Table 3:** Transmitted drug resistance mutation and confer resistance to ART drugs among the ART treatment naïve.

Samples	PI SDRMs	NRTI SDRMs	NNRTI SDRMs	Confer resistance to ART drugs
Manipur005	—	Y115F, M184V	K103N, Y181C, G190A	ABC (H), DDI (L), FTC (H), 3TC (H), EFV (H), ETR (I), NVP (I), RPV (H)
Manipur017	I54V, L76V, I84V	K65R, M184V	Y188L	ABC (H), d4T (I), DDI (H), FTC (H), 3TC (H), TDF (I), EFV (H), ETR (L), NVP (H), RPV (H), ATV/r (H), DRV/r (I), FPV/r (H), IDV/r (H), LPV/r (H), NFV (H), SQV/r (H), TPV/r (I)
Manipur021	—	K219Q	—	AZT (L), d4T (L)
Manipur034	—	K219Q	—	AZT (L), d4T (L)
Manipur056	—	M184V, T215F	Y181C	ABC (L), AZT (I), d4T (I), DDI (L), FTC (H), 3TC (H), EFV (I), ETR (I), NVP (H), RPV (I)

SDRMs were analyzed by using the Calibrated Population Resistance (CPR) tool at HIV standard database (http://cpr.stanford.edu/cpr.cgi). SDRMs, surveillance drug resistance mutations; H, high; I, intermediate; L, low.

Among the remaining 30 ART treatment experienced individuals, 15 (50%) individuals harbored HIV-1 drug resistance strains that conferred resistance to NRTIs, NNRTIs and/or PIs (Fig. 3a). The results further showed that 6 (20%), 12 (40%) and 4 (13.3%) individuals had DRMs at the target sites for NRTIs, NNRTIs and PIs respectively (Fig. 3b). Twenty-five different types of ADRMs in fifteen individuals have been defined based on the mutation sites (11 at NRTIs and 14 at NNRTIs target sites) in the reverse transcriptase gene associated with resistance to reverse transcriptase inhibitors and five (one major and four minor) different types of ADRMs in protease gene associated with resistance to protease inhibitors. One sample (Manipur036) harbored multiple drug resistance mutations at NRTIs and NNRTIs sites which confer high and intermediate level of resistance to all known reverse transcriptase inhibitors (Table 4). The common DRMs found among the ART treatment experienced individuals were targeted at (i) NRTI positions: M184V (10%, 3/30), M184I (6.7%, 2/30) and M41L, D67N, T69D, K70G/R, Y115F, T215N and K219R/Q (3.3% each, 1/30) (ii) NNRTI positions: A98G, K103N, V179D/E, Y181C, G190A/R (6.7% each, 2/30), K101E, V106A/M, V108I, Y181F, H221Y and M230I (3.3% each, 1/30) and (iii) protease inhibitors positions: major mutation M46L (3.3%, 1/30) and other mutations L24I, G48K and G73S (3.3% each, 1/30). HIV-1 sequences containing DRMs were widely dispersed in the phylogenetic tree among sequences with no DRM. The results also reveal that 16%, 6%, 6%, 16%, 16%, 16% of the individuals were harbored virus which confer resistance to ABC, AZT, d4T, DDI, FTC, 3TC (NRTIs drugs) respectively while 36%, 23%, 33%, 26% of the individuals were harbored virus which confer resistance to EFV, ETR, NVP and RPV (NNRTIs drugs) respectively (Fig. 3c).

**Table 4:** Drug resistance mutations and confer resistance to ART drugs among the ART treatment experienced individuals.

Samples	PIs	NRTIs	NNRTIs	Confer resistance to ART drugs
Manipur009	—	—	K103N	EFV (H), NVP (H)
Manipur013	M46L	—	—	ATV/r (L), FPV/r (L), IDV/r (L), LPV/r (L), NFV (L), SQV/r (L), TPV/r (L)
Manipur018	—	—	V179E	EFV (L), ETR (L), NVP (L), RPV (L)
Manipur022	—	M41L	V108I	AZT (L), d4T (L), DDI (L), EFV (L), NVP (L)
Manipur023	—		V106A	EFV (I), NVP (H)
Manipur032	—	M184I	G190R	ABC (L), DDI (L), FTC (H), 3TC (H)
Manipur033	—		M230I	NVP (I), RPV (I)
Manipur036	—	K70G, Y115F, M184V, T215N	K103N, Y181C, G190A, H221Y	ABC (H), AZT (L), d4T (I), DDI (I), FTC (H), 3TC (H), TDF (I), EFV (H), ETR (H), NVP (H), RPV (H)
Manipur040	—	D67N, T69D, K70R, M184V	A98G, K101E, V106M, Y181F, G190A	ABC (L), AZT (I), d4T (I), DDI (I), FTC (H), 3TC (H), TDF (L), EFV (H), ETR (I), NVP (H), RPV (H)
Manipur043	G48K	M184I	G190R	ABC (L), DDI (L), FTC (H), 3TC (H)
Manipur046	G73S	—	—	ATV/r (L), FPV/r (L), IDV/r (L), NFV (L), SQV/r (L)
Manipur048	—	—	V179D	EFV (L), ETR (L), NVP (L), RPV (L)
Manipur051	L24I	—	V179E	EFV (L), ETR (L), NVP (L), RPV (L)
Manipur052	—	—	V179D	EFV (L), ETR (L), NVP (L), RPV (L)
Manipur053	—	M184I	A98G, Y181C	ABC (L), DDI (L), FTC (H), 3TC (H), EFV (I), ETR (I), NVP (H), RPV (H)

Drug resistance mutations among the treatment exposed individuals were analyzed by Genotypic Resistance Interpretation Algorithm at the HIVdb program (http://sierra2.stanford.edu/sierra/servlet/JSierra. H, high; I, intermediate; L, low.

### Origin of transmitted drug resistance sequences of Manipur HIV-1

**Figure 4:**
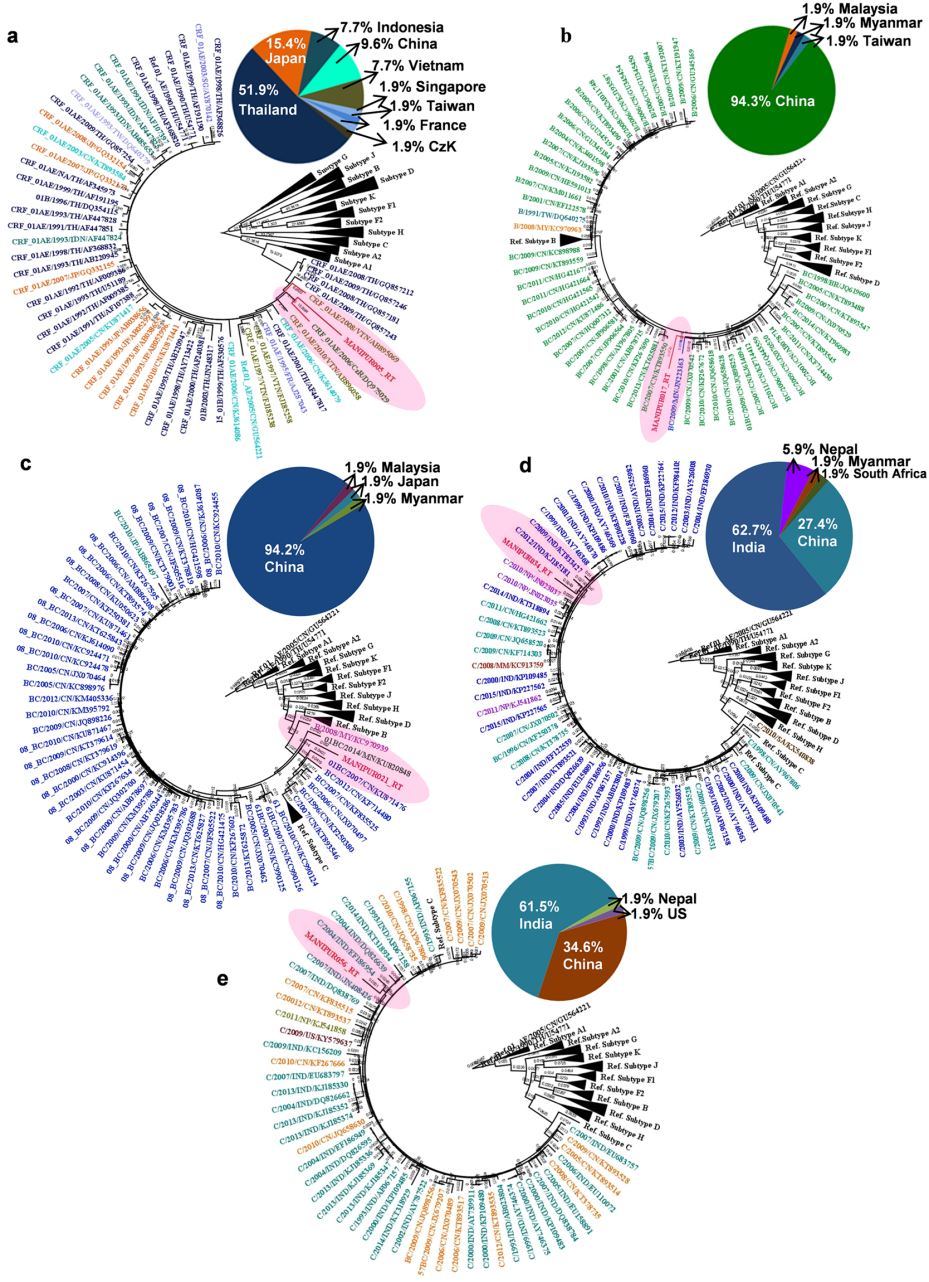
TDRM RT sequences ML phylogenetic tree of HIV-1 Manipur sequences and 52 highly similar (nucleotide similarity >94%) sequences from other countries selected with Blastn analyses. Sample Manipur005_RT (**a**), sample Manipur017_RT (**b**), sample Manipur021_RT (**c**), sample Manipur034_RT (**d**) and sample Manipur056_RT (**e**). Manipur sequences are shown in highlighted red color. The tree was rooted using reference subtype sequences. The numbers along branches correspond to aLRT values. Bar is in nucleotide substitutions per site. The pie chart shows the proportion of countries represented by the Blastn analysis. Countries correspond with defined colors which are specified in each pie chart.

The maximum likelihood (ML) tree of the five TDRM reverse transcriptase sequences is shown in Fig. 4. The strains from Manipur (Manipur005_RT, Manipur017_RT, Manipur021_RT, Manipur034_RT and Manipur056_RT) are highlighted in red colour and those from other countries are indicated with different colour in the taxa. The sequence (Manipur005_RT) is phylogenetically related and branched within high support with CRF_01AE strains isolate from Vietnam and Czech Republic (Europe) (Fig. 4a). The sequence (Manipur017_RT) is branched with the other BC strains from China, but significant positioned within a highly supported monophyletic clade that was composed by BC isolates from Myanmar (Fig. 4b). Manipur021_RT is formed well-supported monophyletic clusters with recombinant BC sequences of Myanmar and China (Fig. 4c). The sequence Manipur034_RT is closely related with all subtype C sequences obtained from India than to the HIV sequence from other countries; China and Nepal (Fig. 4d). The phylogenetic analysis of Manipur056_RT (subtype C) sequence showed a close relationship with subtype C strain from India (Fig. 4e). Overall, the findings show that out of five RT transmitted drug resistance samples, 2 recombinant BC samples namely samples Manipur017_RT and Manipur021_RT were formed close relationship with BC isolate from Myanmar, 2 samples; Manipur034_RT and Manipur056_RT have formed relationship with subtype C isolate from mainland India and 1 sample Manipur005_RT (CRF_01AE) had traced its origin from Vietnam.

## Discussion

Although Manipur has been being one of the hard hit epicenters of the HIV/AIDS epidemic in India since the first detection of HIV-1 in the year 1990, study on drug resistance profile among the HIV infected wives of IDUs of Manipur is limited. Many of HIV/AIDS patients may have been treated inappropriately due to lack of sequence interpretations of newly mutated virus which enhance the viral activity even in presence of antiviral drugs^12^. The surveillance of transmitted and acquired drug resistant strains of HIV-1 is one of the priorities for HIV/AIDS prevention and control. Therefore, this study was conducted to investigate the drug resistance, genetic variant and possible origin of TDR among the HIV-1 infected wives of IDUs in Manipur state. The study population in the current study comprises a representative sample of HIV-1 infected wives of IDUs recruited in the year 2016–2017 from Manipur. The finding reveals 35.7% had Transmitted Drug Resistance Mutations (TDRMs) while 50% had Acquired Drug Resistance Mutations (ADRMs). These TDRMs and ADRMs have the ability to confer resistance to nucleoside reverse transcriptase inhibitors (NRTIs), non-nucleoside reverse transcriptase inhibitors (NNRTIs) and/or protease inhibitors (PIs) drugs which hinder before and during treatment of HIV. When we compared the prevalence of NRTIs, NNRTIs and PIs related mutations in ART naive and ART experienced individuals, the findings of this study suggest that ART naïve individuals had harbored HIV with 35.7% DRMs while 23.3% DRMs among the ART experienced individuals in NRTIs drug target site (Fig. 3b). Nevertheless, In NNRTIs drug target site, 21.4% DRMs among the ART naïve as compared to 40% DRMs in ART experienced individuals (Fig. 3b). While in PIs drug target site, ART naïve individuals has harbored HIV with 7.1% mutation compared to 13.3% among the ART experienced individuals (Fig. 3b). Therefore this indicates that the levels of drug resistance in RT region were high in the Manipur state. Further, M184V was identified to be the most frequent mutation among the ART naïve (21.4%) and ART experienced (10%) individuals conferring resistance to NRTIs. Interestingly, M184V could reduce pathogenicity of HIV, and increase the susceptibility to other drugs targeted at NRTI sites^13,14^. Among the ART naïve patients, most of the HIV-1 sequences have resistance against the anti-viral drugs; stavudine (d4T) while among the treatment experienced samples, resistance against the efavirenz (EFV), nevirapine (NVP) and rilpivirine (RPV) were frequency detected (Fig. 3c). The overall finding of this study has raised the necessity for genotyping the drug resistance mutation to assist the clinicians in selecting the potent antiretroviral drug regimens that will enhance the appropriate treatment responses.

Further, the findings reveal that the majority of the isolated HIV-1 sequences (77.3%) were subtype C while 9.1% was discordant subtype, 6.8% was subtype B, 4.5% was CRF_01AE and 2.3% was URF_BC (Fig. 2e). The prevalence of subtype C in Manipur is almost consistent with previous finding. However, this study also shows the appearance of circulating recombinant form CRF_01AE for the first time after three decade of HIV prevalence in Manipur. The viral CRF_01AE strain was highly prevalent in the neighbouring countries viz China, Myanmar and other south-east Asian countries. Previously Chen R. *et al.*^15^ has reported that CRF01_AE viruses had highly drug resistant RT which significantly influences on HIV-1 mutation frequencies. Therefore, appearance of CRF_01AE for the first time shows the changing molecular epidemiology and evolution of drug resistance strain.

**Figure 5:**
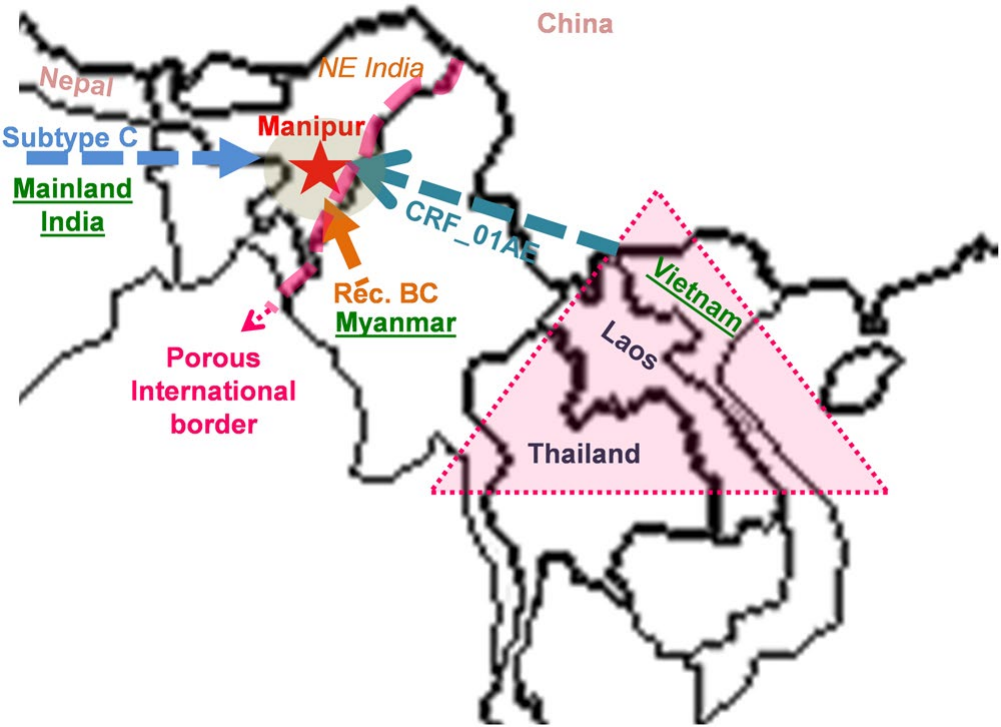
Map of the study site and possible route of origin of transmitted drug resistant strain of Manipur. Red star indicates the study site. Arrows indicate the possible route of HIV origin according to viral subtype and pink triangle indicates the golden triangle area.

Further, we investigated the origin of five HIV-1 transmitted drug resistance strains circulating among the wives of IDUs of Manipur. The BLAST search of Manipur005_RT mainly retrieved CRF_01AE sequences from Thailand (51%), Japan (15%), China (9%) and Indonesia (7%) while the BLAST similarity search of the samples; Manipu017_RT and Manipur021_RT retrieved BC sequences mainly isolated in China (94%). The phylogenetic analysis Manipur034_RT and Manipur056_RT sequence showed a close relationship between Manipur and mainland Indian samples (Fig. 4d,e). The finding is consistent when we analyze four HIV-1 sequences without drug resistance mutation (2 BC samples and 2 subtype C samples) (Supplementary Fig. S3). The URF_BC variant of Manipur was found to be close-associated with BC strain from Myanmar, the subtype C variant was found to be introduced from mainland Indian while the CRF_01AE recombinant has traced its origin from Vietnam. The results suggested that samples which are identified as URF_BC, CRF_01AE and subtype C among the transmitted drug resistant strains were found to be introduced from multiple directions; Myanmar, Vietnam, and mainland India respectively (Fig. 5).

The reason for prevalence of complex HIV-1 genetic variant including CRF_01AE in Manipur may be explained due to the geographic location near the south East Asia. It is also noteworthy to mention that certain unique and unclassified mutations which could impact the ART treatment regime were also detected among the study population. Future research on the identification and characterization of the same could be proved beneficial in constantly monitoring the response of individuals to achieve optimal therapeutic outcome. Nevertheless, the study has some limitation. First, as the study focuses on the recently diagnosed among the wives of IDUs in Manipur, the sample size of the study individuals are limited. Moreover, the numbers of ART naïve individuals are also small as compared to the ART experienced individuals. Second, the study focused on data from the PBMC of blood donors from HIV infected wives of IDUs, the rates of TDRMs reported cannot necessarily be concluded to other populations of HIV infected blood donors. Despite these limitations, the current study of HIV-1 drug resistance mutation, genetic diversity and origin of TDR among the HIV infected wives of IDUs in Manipur shows the appearance of CRF_01AE even though subtype C is most frequently found virus. This is the first report of detecting CRF_01AE in Manipur. The study also reveals the high prevalence of drug resistance mutations to NRTIs and NNRTIs among the HIV-1 infected wives of IDUs in Manipur. HIV drug resistant mutations in these individuals represent a great challenge for the future of the ART program. Therefore, our finding suggested that ART programs and the monitoring system for the effectiveness of ART need to be improved. Patients on ART should be constantly monitored for treatment failure and other classes of ART drugs such as protease inhibitors should be included in the regimen for ART-failed patients after the first-line regimen.

## Material and Methods

### Study design, subjects and inclusion criteria

This community-based cross-sectional study was conducted during August 2016 to September 2017 in Manipur, India. The participants were enrolled depending on their suitability with the inclusion and exclusion criteria. Only the HIV-1 infected wives of IDUs residing in Manipur state were enrolled for this study. Regardless of patient ART status, participants were included in this study. Based on the above mentioned inclusion and exclusion criteria, 56 HIV infected wives of IDUs were finally recruited to obtain the objective of this study. A personal inquiry to the official staffs of Manipur AIDS control Society (MACS) revealed that our sampling size represents more than 10% of the HIV infected wives of Manipur state annually.

### Viral *pol* gene sequencing and genetic characterization

Peripheral Blood Mononuclear Cells (PBMC) from HIV-1 infected individuals were isolated using Ficoll Hypaque Premium (GE Healthcare, USA) density gradient centrifugation method as per manufacturer’s instructions for HIV-1 genotyping. Genomic DNA was extracted from PBMC using the automated system QIAcube, with QIAamp DNA Blood Kit (QIAGEN, Germany) following manufacturer’s manual. The entire protease (PR) and part of the reverse transcriptase (RT) (protease, PR: codons 1–99; reverse transcriptase, RT: codons 1–250) of HIV-1 gene was amplified, purified, and sequenced on ABI PRISM 3130xl Genetic Analyzer (Applied Biosystems, Foster City, CA, USA). All original sequence fragments were assembled, edited, and aligned as previously described^16^.

HIV-1 subtypes were determined by REGA HIV-1 Subtyping Tool 3.0 software/jpHMM Program (http://jphmm.gobics.de/submissionhiv.html), Context-based Modeling for Expeditious Typing (COMET, https://comet.lih.lu/)^17–20^ and phylogenetic tree with HIV-1 reference sequences of subtypes A–D, F–H, J, K and CRF_01AE retrieved from the Los Alamos HIV database (www.hiv.lanl.gov). Reference CPZ sequences were also retrieved from HIV database and used as outgroup in the phylogenetic tree analysis. The phylogenetic analyses were performed with the bulk of sequences using the neighbor-joining model based on the Kimura 2-parameter distance matrix and substitution includes transition + transversion using MEGA software (Version7.0)^21^. Tree topology was tested by bootstrap analysis with 500 replicates. The recombination structures were further detected using recombination identification program (RIP)^22^ and confirmed by bootstrapping in Simplot 3.5.1 software^23^.

### Transmitted and acquired drug resistance analysis of HIV-1

The Transmitted Drug Resistance Mutations (TDRMs) and Acquired Drug Resistance Mutations (ADRMs) were identified using the Calibrated Population Resistance (CPR) tool (http://cpr.stanford.edu/cpr.cgi)^24^ and the Genotypic Resistance Interpretation Algorithm of the HIVdb program (http://sierra2.stanford.edu/sierra/servlet/JSierra) respectively^25,26^, both available through the Stanford University HIV Drug Resistance Database. In accordance to the World Health Organization (WHO) guidelines, the presence of one or more major resistance mutations to any drug class in treatment-naïve patients was considered as TDRM. The HIVdb program was also used to infer the resistance profile of the HIV-1 sequences. The level of resistivity against antiretroviral drugs; protease inhibitors (PI), nucleoside and non-nucleoside reverse transcriptase inhibitors (NRTI and NNRTI respectively) was estimated according to the HIVdb Interpretation Algorithm version 6.0.11 (Stanford University, Palo Alto, CA, USA)^27^.

### Determination of the origin of TDRMs HIV-1 variants of Manipur

The determination of most probable geographical origin of the transmitted drug resistance genetic variant circulating in Manipur was performed as described by Mendoza Y *et.al*.^28^. Each TDRM Manipur sequence was aligned with the 52 HIV-1 sequences isolated world-wide with the highest Basic local alignment search tool (BLAST) search (http://www.hiv.lanl.gov/content/sequence/BASIC_BLAST/basic_blast.html) similarity score (>94) using clustal W and subject to Maximum Likelihood (ML) phylogenetic analysis using the GTR + I + Γ nucleotide substitution model. The ML tree was reconstructed with the PhyML program^29^ using an online web server (https://www.hiv.lanl.gov/content /sequence/PHYML/interface.html). Heuristic tree search was performed using the SPR branch-swapping algorithm and the reliability of the obtained topology was estimated with the approximate likelihood-ratio test (aLRT) based on the Shimodaira-Hasegawa-like procedure^30^. The ML trees were visualized using the FigTree v1.4.3 program (http://tree.bio.ed.ac.uk/software/figtree/).

### Ethical approval and inform consent

The study protocol was reviewed and approved by the Institutional Human Ethical Committee (IHEC), Manipur University in 2016 (Ref. No: Ac/IHEC/MU/107/2016 dated 19^th^ July 2016). Participants signed written informed consent to participate in this study. This study was conducted in accordance with the Declaration of Helsinki.

## Electronic Supplementary Material

Supplementary Information

## Data Availability

HIV-1 *pol* (Protease and reverse transcriptase) nucleotide sequences analyzed in this study are available in the NCBI database under the GeneBank accession numbers MG251453-MG251529.
