# Supplementary material for: Antiretroviral resistance, genotypic characterization and origin of Human Immunodeficiency Virus among the infected wives of Intravenous drug users in Manipur
Source: Sci Rep. 2018 Oct 12;8:15183. doi: 10.1038/s41598-018-33636-z (PMC6185977; doi:10.1038/s41598-018-33636-z)
Supplement: Supplementary file 1 — Supplementary Information [file 41598_2018_33636_MOESM1_ESM.pdf]

Supplementary Information

Antiretroviral resistance, genotypic characterization and origin of Human Immunodeficiency Virus among the infected wives of Intravenous drug users in Manipur

Adhikarimayum Lakhikumar Sharma[1], Thiyam Ramsing Singh[1] and Lisam Shanjukumar Singh[1]*

[1]Cancer and Molecular Biology Division, Department of Biotechnology, Manipur University, Canchipur- 795003, Manipur, India

Corresponding Author

Correspondence to Lisam Shanjukumar Singh, Department of Biotechnology, Manipur University, Canchipur-795003, Manipur, India, Tel: +91 9615533300; fax: +91 385 2435145; e-mail: shanju.lisam@manipuruniv.ac.in.

Table of Content

Supplementary	Details	Page no.
**Table S1**	Additional sample details	2-3
**Figure S1**	jpHMM results of sequences with TDRMs	4
**Figure S2**	Bootscan analysis	5
**Figure S3**	ML phylogenetic tree analysis of HIV-1 sequences without drug resistance mutation	6

Table S1. Additional sample details

Sample ID	Education	Employment	ART status	HIV Diagnose year
Manipur001	Graduate	Private	Naïve	2015
Manipur002	Graduate	Private	Exposed	2015
Manipur003	Under 10	Private	Exposed	2016
Manipur004	Under10	Unemployment	Exposed	2016
Manipur005	Graduate	Private	Naïve	2015
Manipur006	Graduate	Unemployment	Exposed	2015
Manipur007	Graduate	Unemployment	Exposed	2014
Manipur008	Graduate	Unemployment	Exposed	2015
Manipur009	Graduate	Govt. Employee	Exposed	2014
Manipur010	Graduate	Unemployment	Exposed	2016
Manipur011	Under 10	Govt. Employee	Naïve	2016
Manipur012	Graduate	Unemployment	Exposed	2014
Manipur013	Graduate	Unemployment	Exposed	2014
Manipur014	Under 10	Unemployment	Exposed	2015
Manipur015	Graduate	Unemployment	Exposed	2015
Manipur016	Graduate	Private	Exposed	2015
Manipur017	Under 10	Private	Naïve	2015
Manipur018	Graduate	Private	Exposed	2016
Manipur019	Graduate	Unemployment	Exposed	2016
Manipur020	Under 10	Unemployment	Naïve	2015
Manipur021	Graduate	Unemployment	Naïve	2016
Manipur022	Under 10	Student	Exposed	2016
Manipur023	Under 10	Unemployment	Exposed	2014
Manipur024	Graduate	Unemployment	Exposed	2015
Manipur025	Under 10	Unemployment	Exposed	2015
Manipur026	Under 10	Unemployment	Exposed	2014
Manipur027	Graduate	Unemployment	Exposed	2015
Manipur028	Graduate	Unemployment	Naïve	2016
Manipur029	Graduate	Unemployment	Naïve	2015
Manipur030	Graduate	Unemployment	Naïve	2016
Manipur031	Graduate	Unemployment	Naïve	2015
Manipur032	Graduate	Unemployment	Exposed	2016
Manipur033	Under 10	Govt. Employee	Exposed	2014
Manipur034	Graduate	Unemployment	Naïve	2016
Manipur035	Graduate	Unemployment	Naïve	2015
Manipur036	Graduate	Unemployment	Exposed	2015
Manipur037	Graduate	Govt. Employee	Naïve	2016
Manipur038	Under 10	Unemployment	Naïve	2015
Manipur039	Under 10	Govt. Employee	Naïve	2016
Manipur040	Under 10	Unemployment	Exposed	2014
Manipur041	Under 10	Unemployment	Naïve	2016
Manipur042	Graduate	Unemployment	Exposed	2014

Manipur043	Under 10	Unemployment	Exposed	2016
Manipur044	Graduate	Unemployment	Exposed	2015
Manipur045	Graduate	Private	Exposed	2016
Manipur046	Graduate	Private	Exposed	2016
Manipur047	Graduate	Unemployment	Exposed	2015
Manipur048	Graduate	Private	Exposed	2016
Manipur049	Graduate	Unemployment	Exposed	2016
Manipur050	Graduate	Unemployment	Naïve	2015
Manipur051	Under 10	Unemployment	Exposed	2014
Manipur052	Graduate	Unemployment	Exposed	2016
Manipur053	Under 10	Unemployment	Exposed	2015
Manipur054	Under 10	Unemployment	Exposed	2015
Manipur055	Under 10	Unemployment	Exposed	2015
Manipur056	Under 10	Unemployment	Naïve	2016

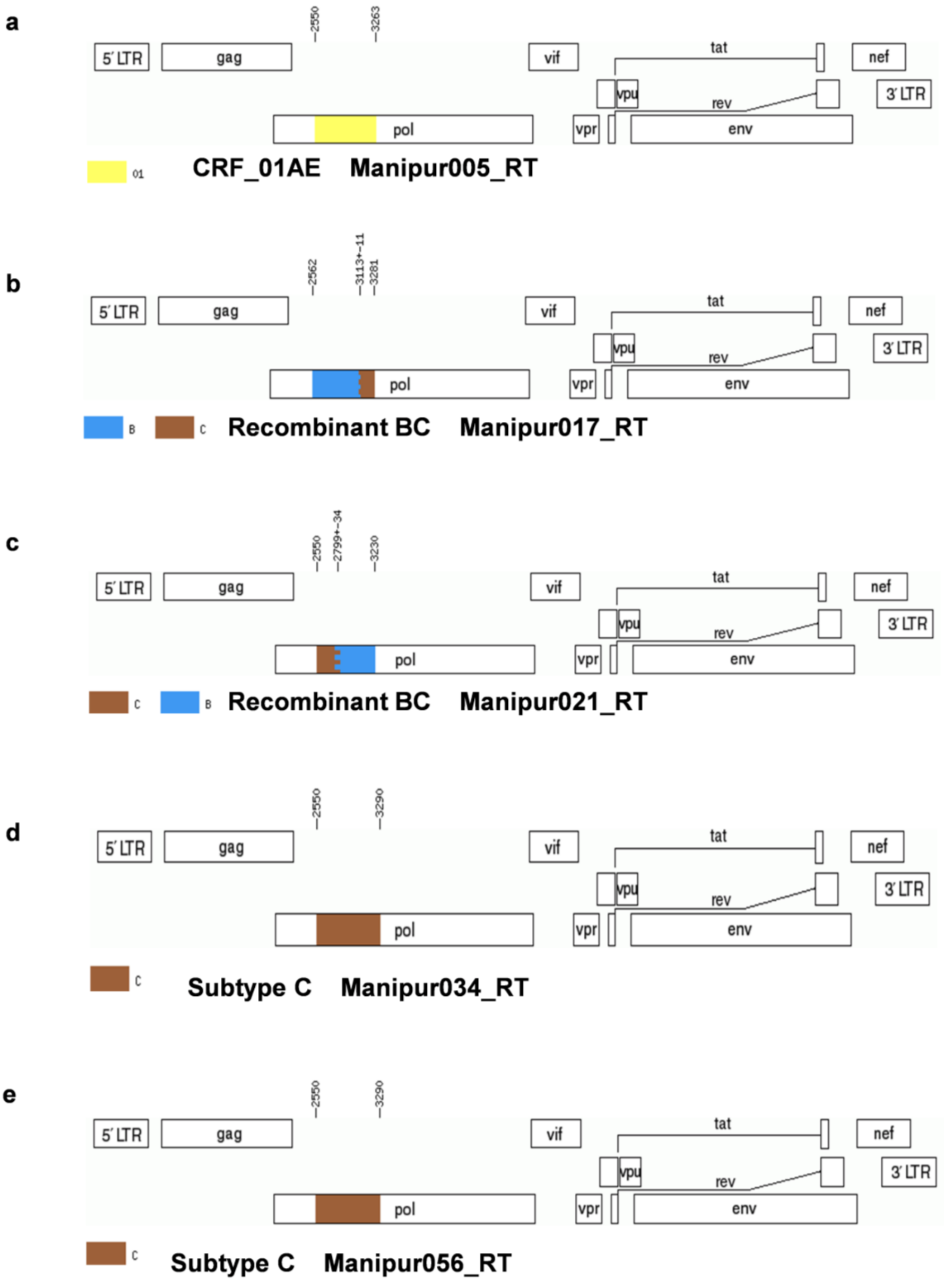

Figure S1. Putative TDRM sequences were submitted to the online implementation of jpHMM at the GOBICS server to determine subtype classifications, breakpoint locations and 95% confidence intervals. Breakpoint locations and confidence intervals are marked on each plot and are equivalent to HXB2 numbering. Manipur005_RT (a); Manipur017_RT (b); Manipur021_RT (c); Manipur034_RT (d) and Manipur056_RT (e).

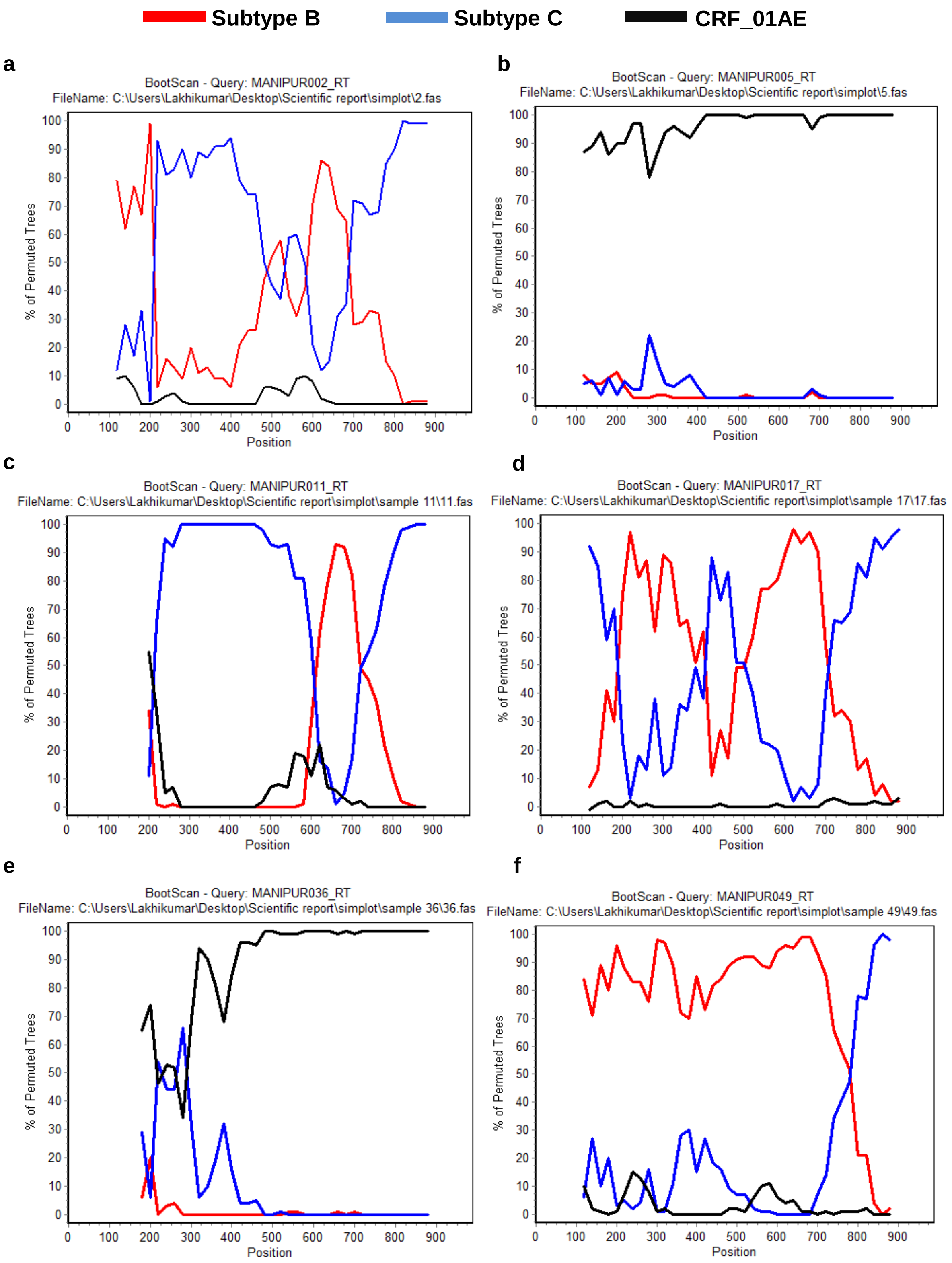

Figure S2. Bootscan illustrations from 6 representatives HIV-1 samples; Manipur002_RT (a), Manipur005_RT (b), Manipur011_RT (c), Manipur017_RT (d), Manipur036_RT (e) and Manipur049_RT (f) queried against Subtype B, Subtype C and CRF_01AE. Bootscan analysis was performed using SimPlot 3.5.1 software configured with 100 bootstrap replicates, a 200 bp window, and a step size of 20 bp. The x-axis shows all aligned nucleotide of the sequence analyzed. The y-axis shows the bootstrap value.

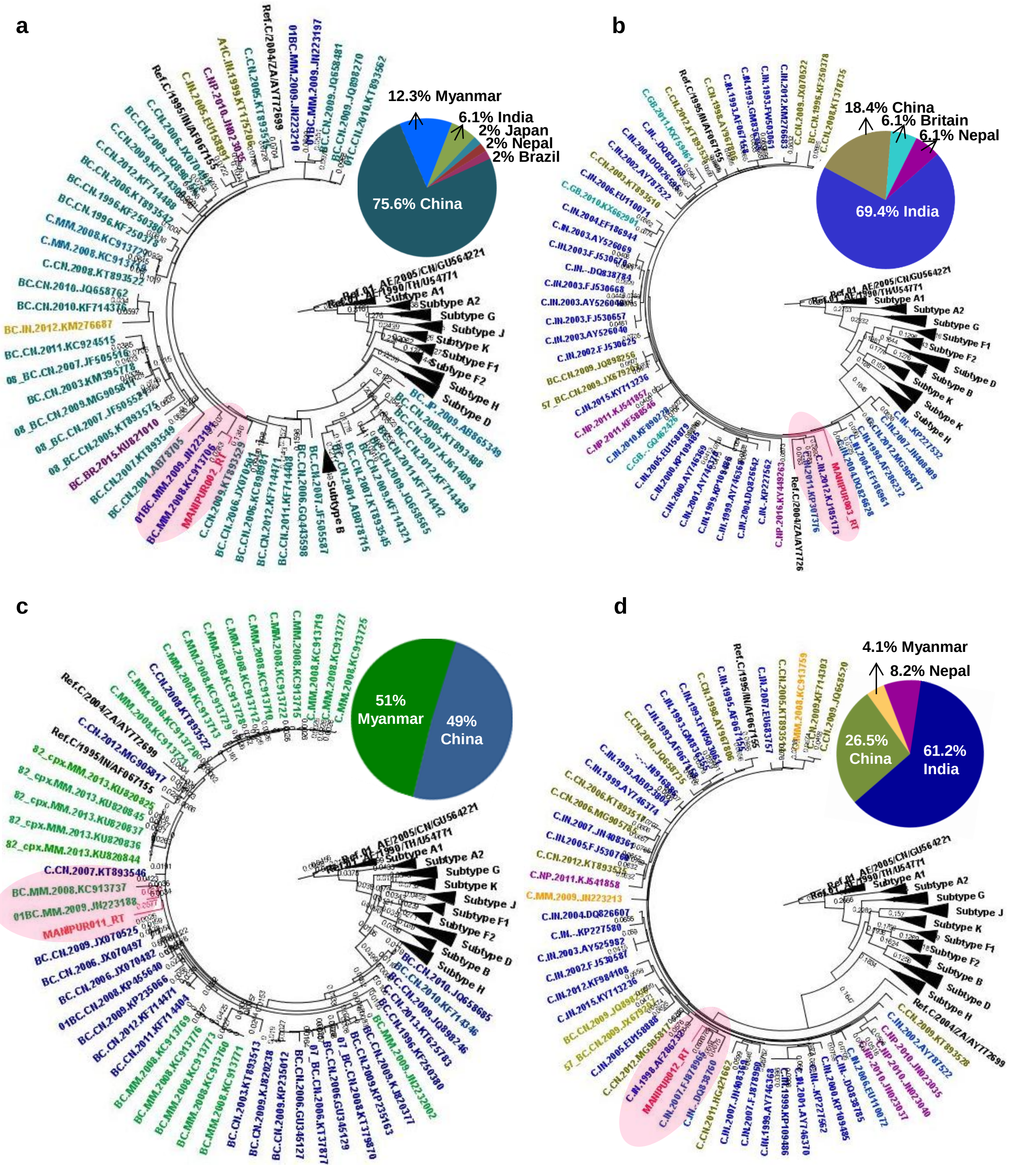

Figure S3. ML phylogenetic tree analysis from 4 representative HIV-1 sequence without drug resistance mutation (2 BC samples and 2 subtype C samples) with highly similar (nucleotide similarity> 94%) sequences from other countries. Manipur002_RT (a), Manipur003_RT (b), sample Manipur011_RT (c), and Manipur012_RT (d). Manipur sequences are shown in highlighted red color. The tree was rooted using reference subtype sequences. The numbers along branches correspond to aLRT values. Bar is in nucleotide substitutions per site. The pie chart shows the proportion of countries represented by the Blastn analysis. Countries correspond with defined colors which are specified in each pie chart.
